# Study on Acoustic Metamaterial Unit Cells: Acoustic Absorption Characteristics of Novel Tortuously Perforated Helmholtz Resonator with Consideration of Elongated Acoustic Propagation Paths

**DOI:** 10.3390/ma18173930

**Published:** 2025-08-22

**Authors:** Yizhe Huang, Qiyuan Fan, Xiao Wang, Ziyi Liu, Yuanyuan Shi, Chengwen Liu

**Affiliations:** 1 Hubei Key Laboratory of Modern Manufacturing Quality Engineering, School of Mechanical Engineering, Hubei University of Technology, Wuhan 430068, China; 102310147@hbut.edu.cn (Q.F.); 102310162@hbut.edu.cn (X.W.); 102410144@hbut.edu.cn (Z.L.); 2 Dongfeng Liuzhou Motor Co., Ltd., Liuzhou 545005, China; 3 State Key Laboratory of Digital Manufacturing Equipment and Technology, Huazhong University of Science and Technology, Wuhan 430074, China; d201980200@hust.edu.cn

**Keywords:** acoustic metamaterial unit cell, novel Helmholtz muffler, perforated and tortuous characteristics, sound wave propagation path, muffler sound transmission loss (STL), low frequency broadband

## Abstract

Traditional sound-absorbing materials, which are intended to address the issue of low-frequency noise control in automobile air-conditioning duct mufflers, have limited noise reduction effects in small spaces. Because of their straightforward structure and excellent controllability, acoustic metamaterials—particularly Helmholtz resonators—have emerged as a research hotspot in low-frequency noise reduction. However, existing technologies have issues such as restricted structural scale, narrow absorption frequency bands, and conflicts with ventilation requirements. To address these, this paper proposes a new type of Helmholtz perforated and tortuous-characteristic duct muffler for the unit cell of acoustic metamaterials. Through the innovative structural design combining a perforated panel with a multi-channel tortuous cavity, the length of the channel is changed in a limited space, thereby extending the sound wave propagation path and enhancing the dissipation of sound wave energy. Meanwhile, for the muffler, acoustic theoretical modeling, finite element simulation, and parametric optimization methods are adopted to systematically analyze the influence of its key structural parameters on the sound transmission loss (STL) of the muffler. Compared with the traditional folded-channel metamaterial, the two differ in resonance frequency by 38 Hz, in transmission loss by 1.157 dB, and in effective bandwidth by 1 Hz. This research provides theoretical support and design basis for solving the problem of low-frequency noise control in ventilation ducts, improves low-frequency broadband sound absorption performance, and promotes the engineering application of high-efficiency noise reduction devices.

## 1. Introduction

The noise control of automotive air-conditioning ducts is of great significance for improving vehicle sound quality. In the air-conditioning ventilation duct system, due to the narrow space and the generally low noise frequency, it is difficult for traditional sound-absorbing materials and structures to achieve effective noise reduction without increasing the volume. Therefore, developing new low-frequency noise reduction materials with compact structures and superior performance has become a key direction in current research.

Acoustic metamaterials are a class of artificially structured materials with unconventional physical properties, possessing acoustic characteristics not found in ordinary materials, such as negative mass density, negative equivalent elastic modulus, and negative refractive index. This material typically has three core characteristics: (1) its structure is artificially designed and constructed into composite units with specific functions; (2) the physical properties it exhibits are not possessed by conventional materials in nature; (3) its macroscopic acoustic properties depend mainly on the configuration rather than the inherent properties of the constituent materials. In recent years, these materials have become a research hotspot in the field of acoustic noise reduction [1,2,3]. In 2000, Liu et al. [4] first introduced the concept of “acoustic metamaterials” and proposed phononic crystals based on local resonance, which can achieve strong reflection of 400 Hz sound waves. With a thickness of only 2 cm, they can break through the traditional sound insulation limit. In 2002, Liu et al. [5] further explored three-component composite structural acoustic metamaterials, using multiple scattering theory to regulate resonant bandgaps and Bragg bandgaps, realizing precise control over the propagation characteristics of elastic waves. In 2007, Ding et al. [6] proposed a zinc blende-structured double-negative acoustic metamaterial which can simultaneously exhibit negative bulk modulus, negative mass density, and negative Poisson’s ratio near the resonant frequency, demonstrating typical resonant metamaterial properties. In the same year, Ambati et al. [7] studied surface resonance states in acoustic metamaterials and proposed the concept of an “acoustic superlens”, bringing new directions to ultrasonic imaging technology. In 2009, Liang et al. [8] designed a one-dimensional acoustic diode structure composed of a superlattice coupled with a strongly nonlinear medium, and verified the application potential of metamaterials in asymmetric wave transmission through numerical simulations. In 2015, Popa et al. [9] proposed active acoustic metamaterials whose responses can be controlled in real time by digital circuits, showing the potential to break through the diffraction limit in terms of lenses, beam imaging, and harmonic functions. In the same year, Shen et al. [10] constructed a broadband hyperbolic acoustic metamaterial. Through multiple groups of thin plate arrays oriented in the y-direction, it exhibited negative density and hyperbolic dispersion at low frequencies, realizing subwavelength focusing and excellent low-frequency sound absorption capacity. Dario [11] investigated the vibro-acoustic properties of square unit cells of porous acoustic materials containing circular inclusions using the shifted unit technique. The proposed design of embedding periodic inclusions into the porous layer has a significant effect in improving acoustic performance (e.g., transmission loss). Magliacano et al. [12] employed the displacement unit technique to study the dispersion characteristics of periodic porous materials under the equivalent fluid model and their correlation with acoustic properties, and found that the equivalent transmission loss curves obtained from eigenvalue analysis are in good agreement with the results of classical methods.

Among various acoustic metamaterials in the field of noise reduction, Helmholtz resonators stand out as one of the most representative types due to their simple structure, clear frequency response, and low cost, exhibiting excellent performance in low-frequency noise control. In 2005, Tang [13] conducted research on Helmholtz resonators with conical necks, revealing that a larger cone angle and a larger cavity lead to more significant low-frequency absorption effects. Wang et al. [14] proposed a new type of metamaterial combining Helmholtz resonant cavities with porous material modulation crowns, enabling ultra-broadband and high-efficiency sound absorption. Ryoo et al. [15] paralleled microperforated plates with Helmholtz resonant cavities, achieving 90% absorption in the range of 380–790 Hz. Wang et al. [16] designed a combined structure of microperforated plates and extended-neck resonators through optimization algorithms, realizing broadband and high-efficiency absorption of noise below 500 Hz in a small area. Additionally, Yaw et al. [17] introduced rough-neck structures, achieving strong absorption in the low-frequency range below 200 Hz; Mei et al. [18] designed segmented Helmholtz metamaterials with embedded necks, attaining over 90% sound absorption efficiency in the range of 450–1750 Hz. Chen et al. [19] proposed a compact side-slit Helmholtz resonator, with an absorption coefficient exceeding 0.8 in the range of 470–930 Hz, making it suitable for practical application scenarios. In 2024, Zhang et al. [20] developed ventilated Helmholtz acoustic metamaterials, where the double-layer structure achieves nearly perfect absorption at approximately 687 Hz, and the four-layer structure exhibits broadband performance in the range of 454–698 Hz. Domingo-Roca et al. [21] combined membrane structures to realize directional low-frequency sound absorption, expanding the application potential in open environments.

In addition to the optimization of structural units themselves, research on introducing Helmholtz resonant structures into pipeline mufflers has also achieved significant results. Wu et al. [22] proposed a new muffler design method that combines the advantages of quarter-wavelength tubes and Helmholtz mufflers, and verified that their method features a wider noise elimination frequency band and a smaller radial size. Nguyen et al. [23] designed a double-layer slit structure, achieving 30 dB noise reduction in the range of 480–950 Hz. Shao et al. [24] proposed a muffler with a membrane structure and multiple Helmholtz cavities, and confirmed that the noise elimination frequency can be adjusted by changing the number of membranes. Liu et al. [25] proposed a new metamaterial structure combining multi-sized Helmholtz cavities with porous materials, achieving excellent sound absorption performance in the 250–1600 Hz frequency band. Wu et al. [26] focused on the control of air duct modulation noise, verifying significant noise reduction effects at the first four blade frequencies. Cho and Lee [27] constructed a multi-unit combined structure with the help of generative design technology, meeting the overall noise reduction target of 25 dB in the 100–6000 Hz frequency band. Furthermore, Gao et al. [28] realized a frequency-tunable Helmholtz muffler by regulating the cavity volume with a stepping motor, which is adapted to rectangular ventilation systems.

In conclusion, although acoustic metamaterials based on Helmholtz resonators have made significant progress in the field of low-frequency noise reduction, they suffer from three common issues: insufficient resonance paths when structural dimensions are limited, making it difficult to achieve effective low-frequency absorption; a single resonance peak resulting in a narrow absorption frequency band, which fails to cover actual broadband noise; and conflicts between structural design and ventilation requirements, restricting applications in duct systems. This study suggests a novel kind of perforated tortuous-characteristic metamaterial duct muffler based on Helmholtz to overcome these problems. It improves low-frequency broadband sound absorption performance, increases energy dissipation, and extends the sound wave propagation path inside a constrained space by introducing a structural form that combines a perforated panel with a multi-channel tortuous cavity. This study provides theoretical support and a design basis for the engineering application of high-efficiency low-frequency noise reduction devices by systematically investigating the impact of important structural parameters on transmission loss through the combination of acoustic theoretical modeling, finite element simulation, and parametric optimization.

## 2. Acoustic Theoretical Modeling of Perforated Tortuous-Characteristic Metamaterial Mufflers

**Figure 1:**
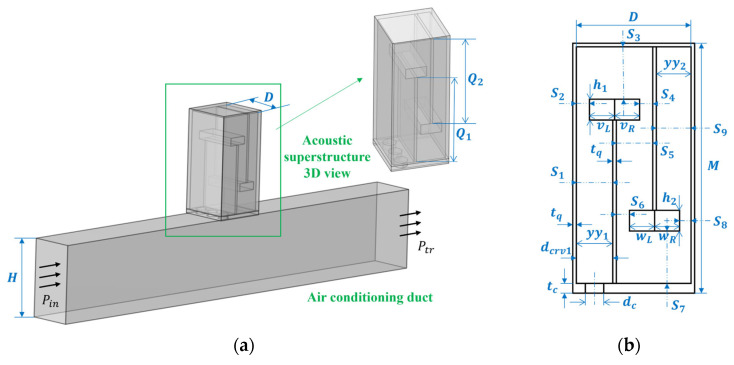
Perforated tortuous-characteristic acoustic metamaterial duct muffler: (**a**) schematic diagram; (**b**) cross-sectional view of the perforated tortuous-characteristic acoustic metamaterial structure.

Figure 1 shows a new type of perforated tortuous-characteristic acoustic superstructure, which is composed of a surface perforated plate and a tortuous-characteristic structure, and is installed on the muffling duct. When sound waves pass through the perforated tortuous-characteristic metamaterial structure on the duct, they first enter the perforations of the perforated plate at the inlet, then propagate in a coupled manner into the tortuous cavity, and continue to propagate along the tortuous channels. Each tortuous cavity can be equivalent to a series of multiple tube channels. Throughout this process, frequent refraction and reflection of sound waves occur, and the energy of the sound waves is gradually converted into heat through the friction effect between the inner wall of the structure and the airflow medium for dissipation. This sound energy loss mechanism is the main path through which the Helmholtz resonator achieves energy attenuation. Based on this design concept, sound wave transmission is simulated in the form of an equivalent fluid [12]; on the basis of a fixed structural thickness, the sound wave propagation path can be effectively extended by changing the channel length, thereby obtaining a good effect that is more conducive to absorbing low- to mid-frequency noise.

The Table 1 includes the meanings of all the formulas mentioned below.The acoustic characteristic impedance of the total surface of the entire rigid-backed perforated tortuous acoustic metasurface structure is as follows:

(1) $$Z_{total}=R_{s}+iY_{s}$$

In the above formula, the physical quantities $R_{s}$ and $Y_{s}$ stand for acoustic resistance and acoustic reactance, respectively.

Based on the idea of the acoustical–electrical analogy method, the perforated panel and the tortuous cavity channel are analogous to a series relationship in an electrical circuit, and the total surface acoustic impedance $Z_{total}$ of the structure can be expressed as

(2) $$Z_{total}=Z_{ckb}+Z_{q}^{L1}/\varphi_{0}$$

In the above formula, $Z_{ckb}$ is the acoustic characteristic impedance of the perforated plate structure, $Z_{q}^{L1}$ is the surface acoustic characteristic impedance at the first inlet of the tortuous cavity channel resonator, and $\varphi_{0}$ is the correction coefficient, whose value is $S_{0}/S_{i}$. Here, $S_{0}$ is the cross-sectional area of the perforated panel, and $S_{i}$ is as shown in Figure 1b.

### 2.1. Acoustic Theoretical Modeling of Perforated Plate Structures

**Figure 2:**
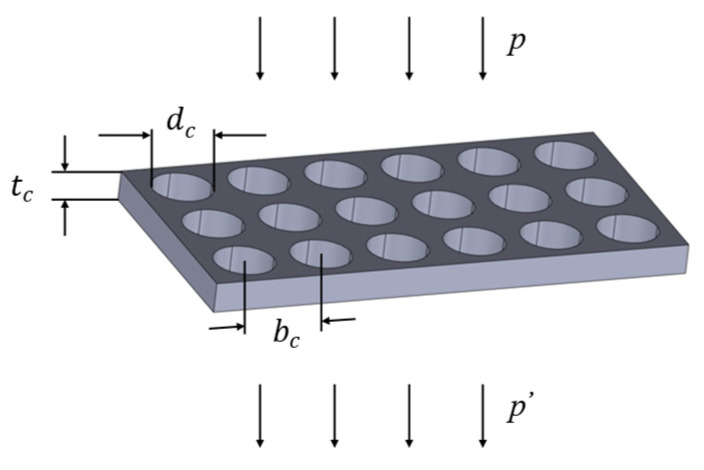
Perforated plate sound absorber.

A perforated plate can be regarded as a structure composed of multiple short tubes connected in parallel. Sound waves propagating inside the short tubes interact with the inner walls of the tubes, and then are converted into heat energy and dissipated due to frictional effects, mainly through the actions of viscosity and heat conduction. On the premise that the tube walls are considered rigid bodies, the micro-vibration of the tube walls themselves will not change the acoustic characteristics of the microtubes, as shown in Figure 2 below.

For the airflow inside the microtube, an increase in the microtube aperture will lead to a corresponding reduction in fluid resistance, whereas a decrease in the microtube aperture will enhance the resistance. When the microtube aperture is reduced to a certain extent, the fluid resistance will increase sharply. If a region is filled with densely arranged fine tube holes, this region has the potential to become a high-performance acoustic material. Based on the transition function [29], the unified calculation formula for the acoustic characteristic impedance $Z_{dck}$ of a single perforation is

(3) $$Z_{dck}=\frac{32\rho_{0}\mu t_{c}}{{d_{c}}^{2}}\sqrt{1+\frac{k^{2}}{32}}+j\omega \rho_{0}t_{c}\left[1+{\left(\sqrt{3^{2}+\frac{k^{2}}{2}}\right)}^{-1}\right]$$

The perforation constant k of the orifice plate is

(4) $$k=k_{0}\frac{d_{c}}{2}=\frac{d_{c}}{2}\sqrt{\frac{\omega \rho_{0}}{\eta }}=\frac{d_{c}}{2}\sqrt{\frac{\omega }{\mu }}$$

In the above formula, $\rho_{0}$ and μ are the density and dynamic viscosity coefficient of air under standard atmospheric pressure (101.325 kPa) and normal temperature (15 °C), with values of 1.226 kg/m^3^ and 1.85 × 10^−5^ Pa·s, respectively; ω is the angular frequency; $t_{c}$ is the central axis length of the micro-short tube; and $d_{c}$ is the aperture.

Based on the idea of the acoustical–electrical analogy method for series and parallel connections [29], the perforated plate is equivalent to a parallel combination of multiple single holes. Thus, the acoustic characteristic impedance $Z_{ckb}$ of the perforated plate structure can be obtained by solving the following equations, (5) to (7), simultaneously:

(5) $$Z_{ckb}=r_{m}+j\omega m_{m}$$

In the above formula, $r_{m}$ and $m_{m}$ are calculated as follows, where $k_{r}$ is the acoustic resistivity constant, $k_{m}$ is the acoustic mass constant, $c_{0}$ is the speed of sound, and σ is the perforation rate of the perforated plate:

(6) $$\left\{\begin{array}{l} r_{m}=\frac{32\mu }{\sigma c_{0}}\frac{t_{c}}{{d_{c}}^{2}}\cdot k_{r}\\ m_{m}=\frac{t_{c}}{\sigma c_{0}}\cdot k_{m} \end{array}\right.$$

(7) $$\left\{\begin{array}{l} k_{r}=\sqrt{1+\frac{k^{2}}{32}}+\frac{\sqrt{2}kd_{c}}{8t_{c}}\\ k_{m}=1+\frac{1}{\sqrt{3^{2}+\frac{k^{2}}{32}}}+0.85\frac{d_{c}}{t_{c}}\\ \sigma =\pi {\left(\frac{d_{c}}{2b_{c}}\right)}^{2} \end{array}\right.$$

### 2.2. Acoustic Theoretical Modeling of Mufflers

For the tortuous channel, its transfer matrix $T_{q}$ is expressed as

(8) $$T_{q}=\left[\begin{array}{cc} 1 & 0\\ 1/Z_{q} & 1 \end{array}\right]$$

Based on the Stinson model [30], calculations are performed using the acoustic transfer matrix method, where the transfer matrix and characteristic impedance can be expressed as

(9) $$T_{q-TMM}=T_{\xi_{l}}T_{bott}$$

(10) $$Z_{q}=T_{q-TMM}\left(1,1\right)/T_{q-TMM}(2,1)$$

For the first term on the right-hand side of Equation (9), $T_{\xi_{l}}$ is the radiation correction matrix from the resonator opening to the waveguide discontinuity, which can be expressed as

(11) $$T_{\xi_{l}}=\left[\begin{array}{cc} 1 & i\xi_{l}k_{0}Z_{0}\\ 0 & 1 \end{array}\right]$$

In the above equation, $k_{0}$ denotes the wavenumber of sound waves in air, $Z_{0}$ represents the acoustic characteristic impedance of air, and $\xi_{l}$ denotes the corresponding radiation correction length, where $d_{crv1}$ represents the inlet width (the width of the i=1 cavity). Based on Mechel’s theory [31], it can be derived as

(12) $$\xi_{l}=0.82\sqrt{Dd_{crv1}}\left[1-0.235\sqrt{\frac{d_{crv1}}{H+M}}-1.32\frac{d_{crv1}}{H+M}+1.54{\left(\frac{d_{crv1}}{H+M}\right)}^{\frac{3}{2}}-0.86{\left(\frac{d_{crv1}}{H+M}\right)}^{2}\right]$$

For the second term on the right-hand side of Equation (9), $T_{bott}$ denotes the acoustic transfer matrix from the opening to the bottom of the resonator cavity:

(13) $$T_{bott}=\left[\begin{array}{cc} \cos(k_{qti}l_{qti}) & iZ_{qti}\sin(k_{qti}l_{qti})\\ i\sin(k_{qti}l_{qti})/Z_{qti} & \cos(k_{qti}l_{qti}) \end{array}\right]$$

Herein, $k_{qti}$ represents the effective wavenumber of the resonator cavity, $l_{qti}$ denotes the approximate effective length of each cavity channel in the tortuous-characteristic acoustic metamaterial structure (this is merely a variable for theoretical derivation; the values of each effective length of the novel metamaterial studied in this paper will be further elaborated upon later in the text), and $Z_{qti}$ stands for the channel characteristic impedance, with its value taken as follows:

(14) $$\begin{array}{l} k_{qti}=\omega \sqrt{\frac{\rho_{cpi}}{K_{cpi}}}\\ Z_{qti}=\sqrt{\rho_{cpi}K_{cpi}} \end{array}$$

Based on thermoacoustic principles, considering viscous losses in the channel, the effective complex density $\rho_{cpi}$ and complex bulk modulus of gas $K_{cpi}$ in the tortuous cavity are calculated via the Stinson model [30], and are given by the following equations:

(15) $$\left\{\begin{array}{l} \rho_{cpi}=\frac{\rho_{0}}{F\left(\psi \right)}\\ K_{cpi}=\frac{\gamma P_{0}}{\left[\gamma -\left(\gamma -1\right)F\left(\psi^{\prime }/\gamma \right)\right]} \end{array}\right.$$

(16) $$\left\{\begin{array}{l} \psi =\mu_{0}/\rho_{0}\\ \psi^{\prime }=\kappa_{0}/\left(\rho_{0}C_{\psi_{0}}\right) \end{array}\right.$$

In Equations (15) and (16), $P_{0}$ is the atmospheric pressure of air under standard conditions, $\mu_{0}=1.48\times 10^{-5}{\;m}^{2}/s$ is the dynamic viscosity coefficient of air at 15 °C and standard atmospheric pressure, γ is the specific heat ratio with a value of 1.4, $\kappa_{0}$ is the thermal conductivity of air with a value of 0.0258 W/(m·K), and $C_{\psi_{0}}$ is the specific heat capacity at constant volume with a numerical value of 0.717 kJ/(kg·K). The function $F\left(x\right)$ is given by

(17) $$F\left(x\right)=1-4{\left(\frac{-j\omega }{x}\right)}^{-\frac{1}{2}}\frac{1}{g_{i}}G\left[\frac{g_{i}}{2}{\left(\frac{-j\omega }{x}\right)}^{\frac{1}{2}}\right]$$

Herein, $g_{i}=\sqrt{\left(4Dd_{crvi}\right)/\pi }$; the zero-order Bessel function and first-order Bessel function are $B_{0}\left(\vartheta \right)$ and $B_{1}\left(\vartheta \right)$, respectively; and the expression for the function $G\left[\vartheta \right]$ is

(18) $$G\left[\vartheta \right]=\frac{B_{1}\left(\vartheta \right)}{B_{0}\left(\vartheta \right)}$$

By consolidating the above derivations, and based on the effective complex density and complex bulk modulus of gas, to determine the surface acoustic characteristic impedance $Z_{q}^{L1}$ at the first inlet of the resonator with tortuous cavity channels in this metamaterial structure, the corresponding impedance for each variation in cross-sectional area and channel length can be calculated via the above impedance transfer method as follows:

(19) $$Z_{q}^{Li}=\left\{\begin{array}{l} -jZ_{qti}\cot\left(k_{qti}l_{qti}\right),i=N\\ Z_{qti+1}\frac{-jZ_{q}^{i+1}\cot\left(k_{qti}l_{qti}\right)+Z_{qti+1}}{Z_{q}^{i+1}-jZ_{qti+1}\cot(k_{qti}l_{qti})},i=N-1,N-2,\ldots ,2,1 \end{array}\right.$$

In the above equation,

(20) $$\begin{array}{l} Z_{q}^{i+1}=Z_{q}^{Li+1}/\phi_{i}\\ \phi_{i}=S_{i+1}/S_{i} \end{array}$$

Herein, $S_{i}$ is the effective cross-sectional area of the cavity channel, N is the number of effective cavity channels, and $l_{qti}$ is the equivalent length of the i-th channel. An exception is the calculation of the impedance $Z_{q}^{LN}$ for the last channel, which is derived using the impedance transfer formula for a rigid-backed termination. It does not account for internal thermoviscous losses and serves as a necessary condition for initiating the recursion of the entire formula. The recursive equation can only be solved when the terminal impedance is known.

Substituting Equations (3) and (19) into Equation (2), the total surface acoustic impedance $Z_{total}$ of the perforated tortuous-characteristic acoustic metamaterial structure can be obtained. $S_{0}$ is the surface area of the perforated plate; when $kS_{0}/D\;$≪1, the formula is as follows:

(21) $$Z_{cr}=\frac{1}{2}\cdot \frac{\rho_{0}c_{0}S_{0}}{H\times D}$$

The sound transmission coefficient τ is given by

(22) $$\tau =1-\frac{Z_{cr}}{Z_{total}}$$

By simultaneously solving Equations (2), (21) and (22), the sound transmission loss (STL) formula for this metamaterial muffler is derived as

(23) $$STL=-20\log_{10}\left(\left|\tau \right|\right)$$

## 3. Simulation Validation of Perforated Tortuous-Characteristic Metamaterial Muffler

### 3.1. Development of a Finite Element Simulation Platform

Based on the perforated tortuous-characteristic superstructure with additional trailing plates proposed in this paper, a geometric model is constructed in Comsol Multiphysics. As shown in Figure 1a, the rectangular structure at the lower part of the model is an air-conditioning duct unit, with the internal cavity cross-sectional dimensions of H × D and a thickness of $t_{c}$; the rectangular structure at the upper part is a unit-cell perforated tortuous-characteristic superstructure, whose internal cavity dimensions are D × D × (M-$t_{q}$ − $t_{c}$). The perforated plate is arranged on the wall of the air duct, and its thickness is the same as that of the air duct wall. A double-baffle design is adopted inside the structure, and the thickness of the baffles is consistent with that of the outer wall, which is conducive to simplifying the analysis.

(1) Domain Division and Physical Field Selection

The noise reduction mechanism of the perforated tortuous-characteristic metamaterial muffler is as follows: Sound waves interact frequently with the walls and partition plates in the micro-holes and tortuous channels, and after multiple reflections and refractions, noise reduction is achieved through thermal dissipation in the cavity. Therefore, in the simulation, “acoustic–solid coupling” is taken as the basis for multi-physics field analysis, and the “thermoviscous acoustics” module is integrated: the air domain in the perforated tortuous channels is set as the thermoviscous acoustics domain (to reflect the viscosity and thermal dissipation caused by abrupt airflow changes in narrow regions), the air domain in the air duct is set as the pressure acoustics domain, and the boundaries of the solid structure are set as the solid mechanics domain.

(2) Boundary Conditions and Loading Methods

In the simulation, solid boundaries are defined as hard acoustic boundaries that neither absorb nor transmit acoustic energy, confining the solution domain to the air regions within the model. The internal cavities of both the perforated tortuous-characteristic metamaterial structure and the duct are designated as air domains, while the interfaces between structural frames are specified as hard acoustic boundaries. Within the pressure acoustics module, one end-face of the duct is configured as a plane wave incidence port ($P_{in}$ in Figure 1a), excited by a 1 Pa amplitude plane wave. The opposite end-face is set as a plane wave transmission port ($P_{tr}$ in Figure 1a). The tortuous-characteristic metamaterial muffler is positioned on one side of the duct, ensuring acoustic waves propagate axially through the duct and enter the structure perpendicularly to facilitate efficient acoustic–structure coupling.

(3) Material Property Definition

Given that the simulation focuses exclusively on the air domain, the pre-defined Air material from COMSOL’s material library was selected, with an ambient temperature of 20 °C. The simulation employs two physics modules: thermoviscous acoustics and pressure acoustics. The key parameters used in the simulation are listed in Table 2 below.

(4) Mesh Generation

**Figure 3:**
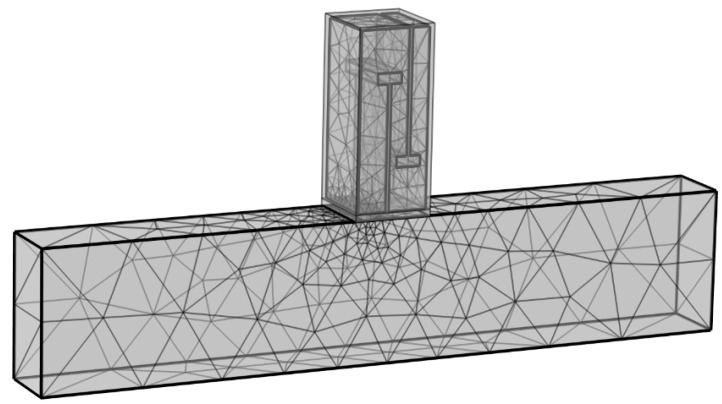
Simulation mesh division diagram of the perforated tortuous-characteristic acoustic metamaterial pipeline muffler.

The air domain is the target of this simulation calculation, and a free tetrahedral mesh is adopted. To guarantee computational accuracy, the maximum element size of the mesh must be smaller than 1/6 of the wavelength corresponding to the highest calculation frequency, while the minimum element size should be smaller than 1/15 of the wavelength corresponding to the lowest calculation frequency. The frequency range studied in this simulation model is approximately 200–700 Hz, and the size ranges of the final mesh elements and the divided meshes are shown in Figure 3 below:

$$\left\{\begin{array}{l} \max\leq 343\left[m/s\right]/f_{up-\lim}\left[\mathrm{Hz}\right]/6=343\left[m/s\right]/700\left[\mathrm{Hz}\right]/6\\ \min\leq 343\left[m/s\right]/f_{low-\lim}\left[\mathrm{Hz}\right]/15=343\left[m/s\right]/200\left[\mathrm{Hz}\right]/15 \end{array}\right.$$

In Comsol Multiphysics, the transmission loss (TL) of the perforated tortuous-characteristic metamaterial muffler can be calculated using the following equation:

(24) $$STL=20\log_{10}\left(\frac{P_{in}}{P_{tr}}\right)$$

In the above formula, $P_{in}$ and $P_{tr}$ represent the incident and transmitted sound pressure at the inlet and outlet positions of the muffler.

### 3.2. Feasibility Verification and Analysis of the Model

In this section, simulations of the perforated tortuous-characteristic metamaterial muffler are performed using the COMSOL Multiphysics 6.1. The obtained transmission loss curves are compared and verified with the theoretical prediction curves to demonstrate the feasibility of the theoretical design of this metamaterial muffler. For the unit-cell double-baffle perforated tortuous-characteristic metamaterial structure, the dimensional parameters of each part are shown in Table 3 below:

The cross-sectional area of the target air-conditioning duct cavity under study is 278.89 cm^2^. However, for the isolated single-cell model, the effective cross-sectional area contributing to noise reduction is only a fraction of the total cavity area. Given that the cross-sectional area of the single-cell cavity is 4 cm^2^, the corresponding effective duct area for noise reduction is 16.7 cm^2^.

**Figure 4:**
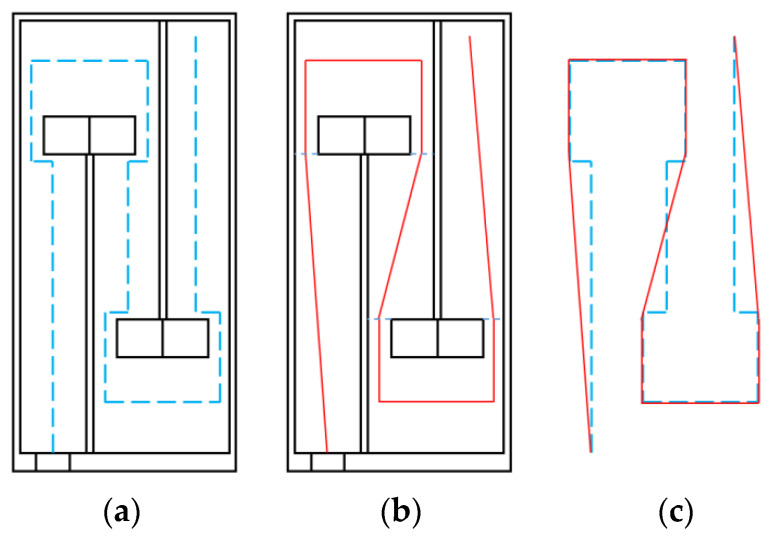
Equivalent path length $l_{qti}$ of the *i*-th channel: (**a**) original equivalent path; (**b**) simplified equivalent path; (**c**) comparison between original and simplified paths.

To derive Equations (19) and (20) in the theoretical model, the equivalent length $l_{qti}$ of the i-th channel and the effective cross-sectional area $S_{i}\;$ of the cavity channel are further explained below. The effective cross-sectional areas $\;S_{i}$ are clearly labeled in Figure 1b. Based on Table 2 (D=2 cm, $t_{c}=2t_{q}=2\;\mathrm{mm}$, M=5.3 cm), the analytical expressions for each $\;S_{i}\;$ are presented in Table 4:

The specific values of each $S_{i}$ obtained from the solution are presented in Table 5 as follows:

Figure 4 shows a cross-sectional schematic of the acoustic metamaterial single cell. During acoustic wave propagation inside the metamaterial single-cell cavity, the cross-sectional area of the propagation path varies significantly, resulting in a more complex effective path—as indicated by the blue dashed line in Figure 4a. To facilitate the calculation of $\;l_{qti}$ and $S_{i}$, the paths around the trailing plates protruding from the end of each baffle are simplified to represent the hypotenuse of a right triangle formed by the original equivalent path. The simplified path is shown as the red solid line in Figure 4b. A comparison of path lengths between the original blue dashed equivalent path and the simplified red solid equivalent path is presented in Figure 4c.

Each segment length of the original equivalent path is labeled, starting from the orifice plate inlet as $l_{q1}$. For the simplified equivalent path, each segment length is labeled starting from the orifice plate inlet as $l_{qt1}$. Based on the triangle inequality principle, it can be derived that

(25) $$\left\{\begin{array}{l} l_{q1}+l_{q2}>l_{qt1}\\ l_{q6}+l_{q7}+l_{q8}>l_{qt5}\\ l_{q12}+l_{q13}>l_{qt9} \end{array}\right.$$

**Figure 5:**
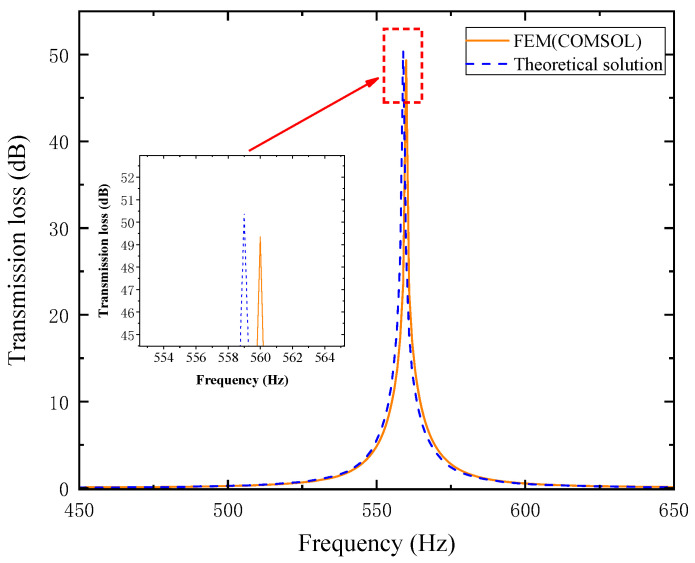
Comparison between simulation and theoretical curves.

Thus, according to Equation (25), the total length of the simplified equivalent path $l_{qt-all}$ is smaller than the total length of the original path $l_{q-all}.$ Based on Table 2, for the single-cell dual-baffle perforated tortuous-characteristic metamaterial structure, the specific analytical expressions for the equivalent path length $l_{qti}$ of each channel are presented in Table 6 as follows:

The specific values of the equivalent path length and total length of each channel obtained through calculation are shown in Table 7 as follows:

Based on the calculation results in Table 6, the total equivalent path length $l_{qt-all}$ in the theoretical analysis is determined to be 144.85 mm. Using both simulation data and theoretical analytical solutions, the transmission loss (TL) curves of the dual-baffle perforated tortuous-characteristic metamaterial structure model were obtained. The plotted curves are shown in Figure 5 below. In the figure, the orange solid line represents the simulation curve, and the blue dashed line represents the theoretical curve. A comparison between the simulation and theoretical curves is presented as follows:

As observed in Figure 5, the theoretical and simulation curves show good agreement. In the simulation, the structure resonates at approximately 560 Hz, with a peak transmission loss (TL) of 49.346 dB. The theoretically calculated TL peak is slightly shifted leftward and marginally higher, reaching 50.395 dB at 559 Hz. The resonance frequency error between the two is 0.178%, and the TL peak error is 2.125%. The errors primarily stem from the following: Acoustic radiation reactance exists at the corners where the cross-sectional area of the metamaterial channels changes. The effective path length of the actual wider channels is slightly shorter than the theoretical value calculated in Table 6; specifically, the total effective channel length $l_{q-all}$ in the theoretical model (Table 5) is slightly longer than the actual length. This results in a slight left shift of the theoretical curve and a slightly higher TL value, which is consistent with practical scenarios. These findings verify the feasibility of the theoretical design for the single-cell muffler with perforated tortuous-characteristic metamaterial features.

**Figure 6:**
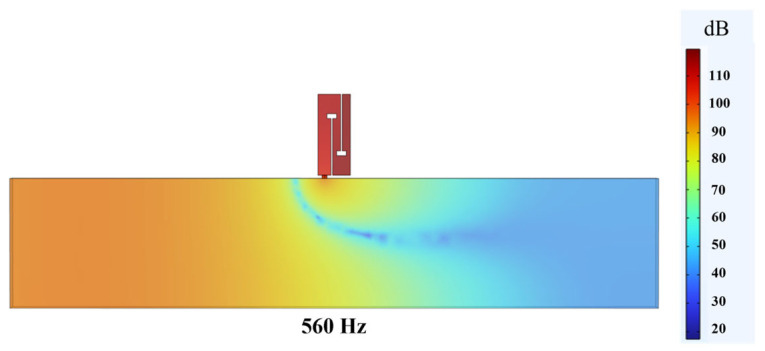
Sound pressure level distribution of the air duct waveguide and the double-baffle perforated tortuous-characteristic metamaterial muffler.

The sound pressure level distribution of the air duct waveguide and the double-baffle perforated tortuous-characteristic metamaterial muffler is shown in Figure 6 below. At the entrance where the air duct leads into the interior of the metamaterial muffler, the sound pressure level drops abruptly to the minimum, enabling effective elimination of sound waves at a frequency of 560 Hz and thus achieving the noise reduction effect.

### 3.3. Analysis on the Effectiveness of Superstructure Design

**Figure 7:**
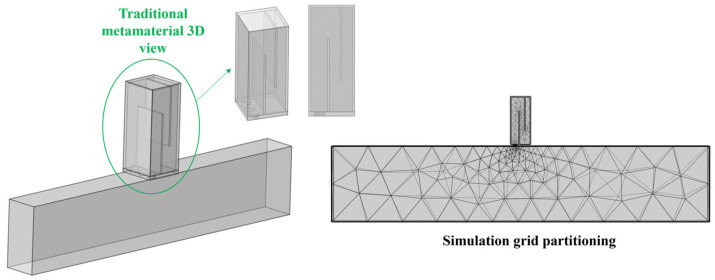
Traditional folded-channel acoustic metamaterial.

For the traditional folded-channel acoustic metamaterials, as shown in Figure 7 below, the main advantage of the superstructure designed in this paper is that, on the basis of a fixed structural thickness and number of partitions, it can effectively extend the sound wave propagation path by adjusting the channel length, thereby achieving a better effect that is more conducive to absorbing mid–low-frequency noise.

For traditional folded-channel acoustic metamaterials, the calculation expression of their equivalent path $l_{cti}$ is as follows:

(26) $$\left\{\begin{array}{l} l_{ct1}=\frac{1}{2}\sqrt{{\left(yy_{1}+t_{q}\right)}^{2}+{\left(M+Q_{1}-t_{q}-t_{c}\right)}^{2}}\\ l_{ct2}=\sqrt{\frac{1}{4}{\left(Q_{1}+Q_{2}\right)}^{2}+{\left(D-yy_{1}-yy_{2}-t_{q}\right)}^{2}}\\ l_{ct3}=\frac{1}{2}\sqrt{{\left(yy_{2}+t_{q}\right)}^{2}+{\left(M+Q_{2}-t_{q}-t_{c}\right)}^{2}}\\ l_{ct-all}=l_{ct1}+l_{ct2}+l_{ct3} \end{array}\right.$$

The specific values of the equivalent path length and total length for each channel of traditional folded-channel acoustic metamaterials obtained through calculation are shown in Table 8 below:

A comparison diagram of the sound transmission loss results between the traditional folded-channel acoustic metamaterial and the perforated tortuous-characteristic metamaterial muffler proposed in this paper is shown as follows.

**Figure 8:**
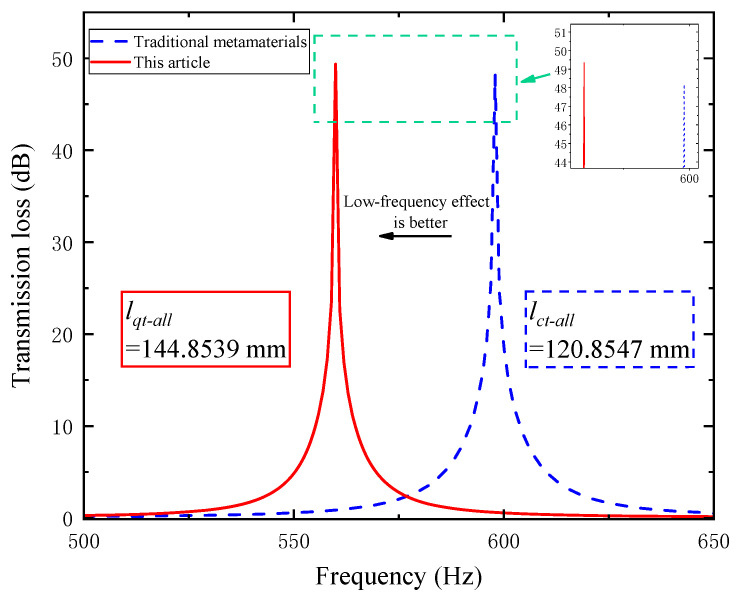
Comparison of transmission loss between traditional metamaterials and the perforated tortuous-characteristic metamaterial muffler proposed in this paper.

As can be seen from Figure 8, there is a significant shift in the peaks of the sound transmission loss curves between traditional metamaterials and the perforated tortuous-characteristic superstructure muffler proposed in this paper: the latter’s peak shifts more toward lower frequencies, resulting in a better low-frequency noise reduction effect. In the simulation, the superstructure proposed in this paper resonates at 560 Hz with a peak sound transmission loss of 49.346 dB, while the resonance peak of the traditional superstructure appears at 598 Hz with a sound transmission loss of 48.189 dB. The difference in resonance frequency between the two is 38 Hz, and the difference in sound transmission loss is 1.157 dB.

**Figure 9:**
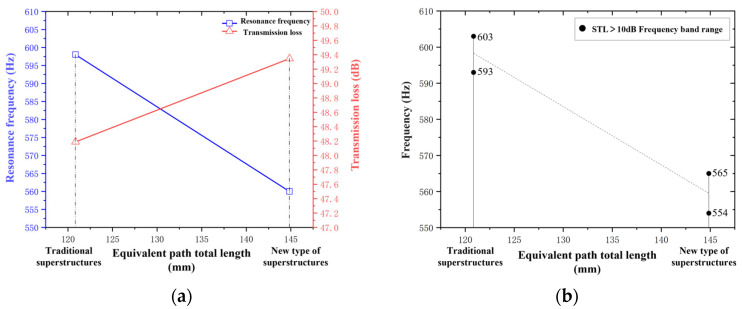
Comparison between traditional and novel perforated tortuous-characteristic acoustic metamaterial mufflers: (**a**) comparison of total equivalent path length and resonance frequency; (**b**) comparison of sound transmission loss.

We define the effective noise reduction frequency band as the range where sound transmission loss (STL) > 10 dB. For the traditional superstructure, the effective bandwidth $\Delta f_{ct}$ is 10 Hz, while the effective bandwidth $\Delta f_{qt}$ of the perforated tortuous-characteristic superstructure proposed in this paper reaches 11 Hz, which is 1 Hz wider than that of the traditional one. The above difference is mainly attributed to the variation in the total equivalent path length of the channels: the total equivalent path length $l_{qt-all}\;$ of the proposed superstructure is 144.8539 mm, whereas that of the traditional superstructure is 120.8547 mm. The comparative relationships between their total equivalent path lengths, resonance frequencies, and sound transmission loss values are shown in Figure 9 below.

As shown in Figure 9 above, the perforated tortuous-characteristic superstructure muffler designed in this paper exhibits a longer equivalent path length, a lower resonance frequency, a higher sound transmission loss, and a wider effective bandwidth of the noise reduction frequency band compared to the traditional superstructure. This is consistent with the design concept of “low-frequency noise reduction” and verifies the effectiveness of the proposed design. The research indicates that when the structural thickness and number of partitions are fixed, adjusting the channel length can effectively improve the low-frequency noise reduction performance of air ducts. This provides a theoretical reference and design basis for the subsequent development of broadband superstructure mufflers.

## 4. Investigation on the Influence of Structural Parameters on the Noise Reduction Performance of Air Ducts

To further explore the influence of regulatory laws of different structural parameters on the sound transmission loss (STL) performance of the muffler in this perforated tortuous-characteristic acoustic metamaterial, this section, based on the unit cell structure of the perforated tortuous-characteristic acoustic metamaterial, investigates the influences of the diameter of holes in the metamaterial’s perforated plate, the number of holes in the perforated plate, the length of internal partitions of the metamaterial, the position of internal partitions of the metamaterial, and the length and thickness of the left and right trailing additional plates of each internal partition of the metamaterial on the noise reduction performance of the air duct.

### 4.1. Influence of Perforation Diameter

**Figure 10:**
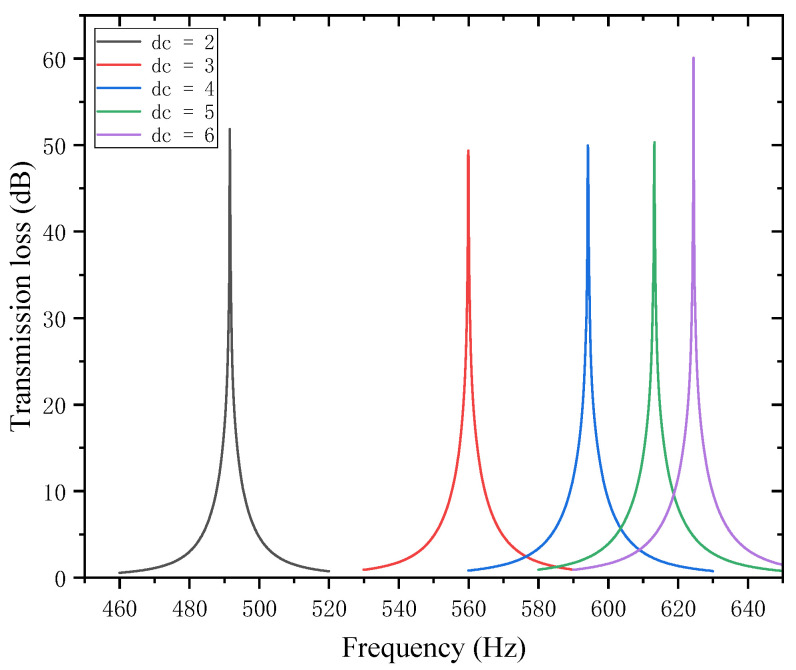
Influence of different perforation diameters on the sound transmission loss of acoustic metamaterial mufflers.

**Figure 11:**
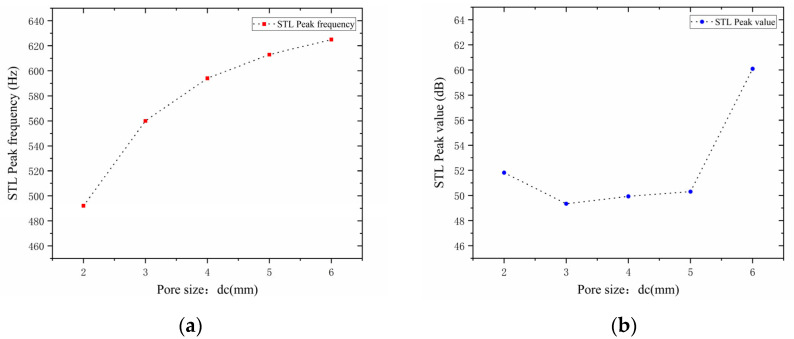
Comparison of noise reduction STL performance under different perforation diameters: (**a**) STL peak frequency; (**b**) STL peak magnitude.

When designing parameters for the perforated tortuous-characteristic acoustic metamaterial targeting specific frequency points or bands, the perforation diameter of the perforated plate is a critical parameter, significantly affecting the sound transmission loss (STL) of duct mufflers. Both noise reduction mechanisms and studies in the literature indicate that with a fixed number of holes, a smaller diameter shifts the STL peak to lower frequencies, while a larger diameter shifts it to higher frequencies. Keeping other structural parameters constant, the STL amplitude–frequency characteristic curves under different hole diameters ($d_{c}$ = 2, 3, 4, 5, 6 mm) are calculated and presented in Figure 10. The STL peak frequency, a key indicator of muffler performance, corresponds to the noise reduction level at that frequency; an increase/decrease in the peak frequency indicates a shift of the STL curve to higher/lower frequencies, with performance comparisons shown in Figure 11.

Analysis of Figure 10 and Figure 11 reveals that the perforation diameter $\;d_{c}\;$ of the perforated plate affects both the STL peak frequency and magnitude. As the perforation diameter increases, the peak frequency of STL shifts to higher frequencies, and the peak magnitude first decreases, then increases, with the turning point occurring at $d_{c}=3\;\mathrm{mm}$. This phenomenon can be explained as follows: a larger perforation diameter allows more sound waves to pass through the perforated plate, leading to more significant energy dissipation and scattering in the high-frequency range, thus shifting the peak frequency toward higher frequencies. In the range of 2–3 mm, increasing the perforation diameter weakens the acoustic damping effect, resulting in a decrease in noise reduction; in the range of 3–5 mm, the resonant noise reduction characteristic becomes prominent, and the noise reduction increases due to the matching of sound waves with the resonant frequency; in the range of 5–6 mm, airflow passes more easily, the acoustic impedance decreases, and the low-frequency resonance effect is enhanced, thereby improving the noise reduction performance.

Further observations reveal that for each 1mm increase in hole diameter, the shifting rate of the peak frequency decreases gradually; that is, a larger hole diameter results in a slower shift toward higher frequencies. The STL peak magnitude decreases slightly in the range of 2–3 mm, increases gently at 3–5 mm, and rises sharply at 5–6 mm. This indicates that the noise reduction effect of the perforated plate is inferior when the diameter is in the intermediate range compared to when it is extremely small or large. Therefore, when designing for target frequencies, it is necessary to reasonably adjust the perforation diameter, balancing both the STL peak frequency and magnitude to achieve optimal noise reduction performance.

### 4.2. Influence of Number of Perforations

**Figure 12:**
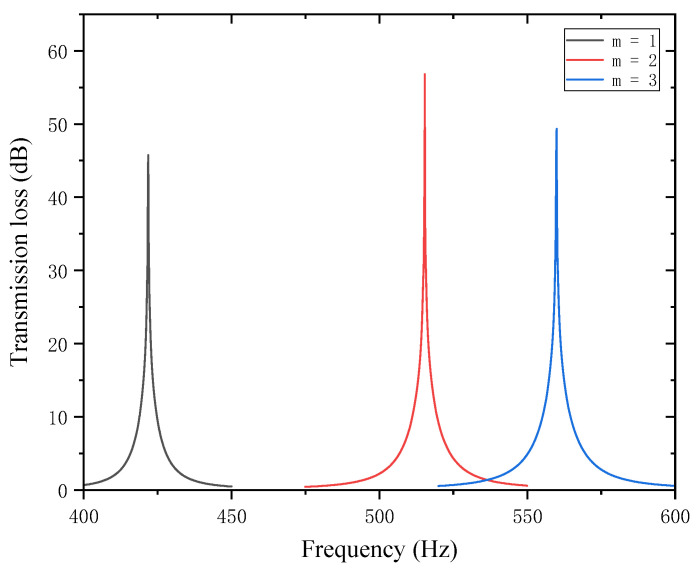
Influence of different numbers of perforations on the sound transmission loss of acoustic metamaterial mufflers.

**Figure 13:**
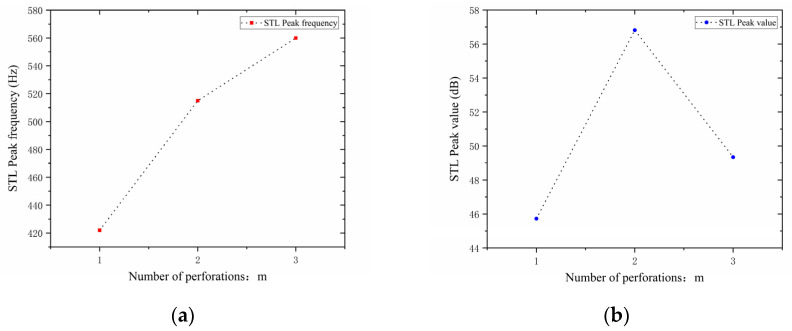
Comparison of noise reduction STL performance under different numbers of perforations: (**a**) STL peak frequency; (**b**) STL peak magnitude.

In the design of the perforated tortuous-characteristic acoustic metamaterial, the number of perforations significantly affects the STL of the muffler. Based on the noise reduction mechanism of perforated plates and the literature experience, it is known that a greater number of perforations m leads to a higher STL peak frequency and a wider bandwidth; conversely, fewer perforations result in a lower peak frequency and a narrower bandwidth. With other structural parameters kept unchanged, the number of perforations m  was set to 1, 2, and 3, and the corresponding sound transmission loss curves of the acoustic metamaterial under these parameters were obtained, as shown in Figure 12. The comparative curves of noise reduction performance are shown in Figure 13.

As can be seen from Figure 12 and Figure 13, the number of perforations m affects both the STL peak frequency and magnitude. With an increase in m, the STL peak frequency shifts toward higher frequencies, while the peak magnitude first increases and then decreases, with the turning point at m=2. When the number of perforations increases, the perforation rate rises, the surface area of the holes and airflow channels increase, the acoustic impedance decreases, and sound transmission increases. This changes the propagation and reflection characteristics of sound waves, leading to an increase in sound transmission loss in high-frequency components and a shift of the resonance frequency toward higher frequencies. When m=1, the obstruction to sound wave propagation is small, resulting in insufficient scattering and interference, thus leading to low sound transmission loss. When m=2, the interference between sound waves through the two holes is enhanced, the standing wave distribution and coupling between holes reach an optimal state, and the sound transmission loss increases sharply. When m=3, the coupling between the acoustic cavity and the folded structure weakens, the standing wave field becomes unstable, the efficiency of acoustic energy dissipation decreases, and the noise reduction amount decreases.

In addition, each time an additional perforation is added to the perforated plate, the growth rate of the peak frequency gradually slows down. The STL peak magnitude increases sharply when changing from a single hole to two holes, but drops sharply when changing from two holes to three holes. This indicates that there exists an optimal matching value for the number of perforations; either too many or too few perforations will significantly reduce the STL peak of noise reduction. Therefore, when setting the number of perforations for target frequencies, comprehensive consideration is required to balance the peak frequency and magnitude, so as to achieve the desired noise reduction performance.

### 4.3. Influence of Internal Partition Length

**Figure 14:**
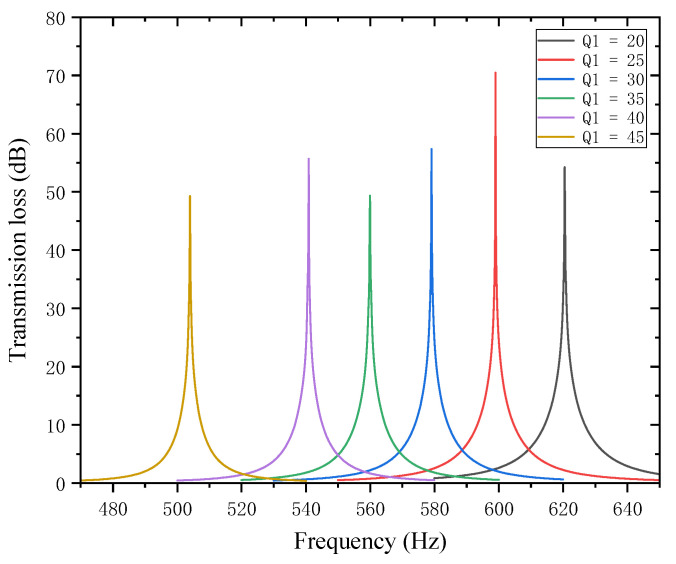
Influence of different lengths of the first partition on the sound transmission loss of acoustic metamaterial mufflers.

**Figure 15:**
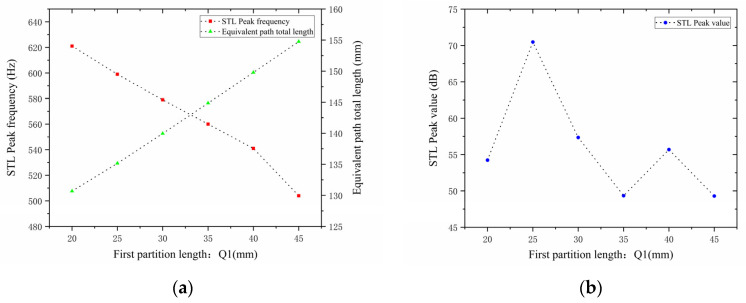
Comparison of noise reduction STL performance under different lengths of the first partition: (**a**) STL peak frequency; (**b**) STL peak magnitude.

The lengths of the two baffles, $Q_{1}$ and $Q_{2}$, are the subject of this paper’s research. By changing the total equivalent path length $l_{qt-all}$ of the cavity channel, changes in partition length influence the noise reduction frequency. They also have an impact on the acoustic impedance, interference patterns, and the path taken by the sound waves, all of which ultimately affect how much noise is reduced. The length of the first partition $Q_{1}$ was set to 20, 25, 30, 35, 40, and 45 mm while maintaining all other parameters constant. Figure 14 displays the corresponding sound transmission loss curves under these conditions, while Figure 15 displays the comparative noise reduction performance curves.

As can be seen from Figure 14 and Figure 15, changes in $Q_{1}$ affect both the STL peak frequency and magnitude. With an increase in $\;Q_{1}$, the total equivalent path length increases, causing the STL peak frequency to shift toward lower frequencies, while the peak magnitude first increases and then decreases, with the turning point at $Q_{1}=25\;\mathrm{mm}$. All peak magnitudes exceed 45 dB. For every 5 mm increase in $Q_{1}$, the increment amplitude of the total equivalent path length gradually increases, and the rate at which the peak frequency shifts toward lower frequencies is slow initially and then accelerates, with a sharp drop in resonance frequency in the 40–45 mm range. The STL peak magnitude increases sharply in the 20–25 mm range, indicating that sound wave interference is most intense and acoustic impedance is maximized in this interval. Therefore, when setting the length of the first partition, the size should be reasonably adjusted to achieve optimal noise reduction performance.

**Figure 16:**
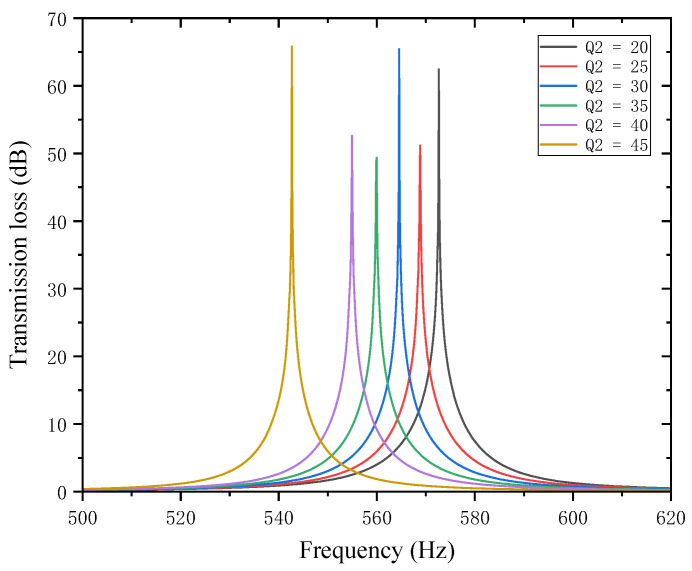
Influence of different lengths of the second partition on the sound transmission loss of acoustic metamaterial mufflers.

**Figure 17:**
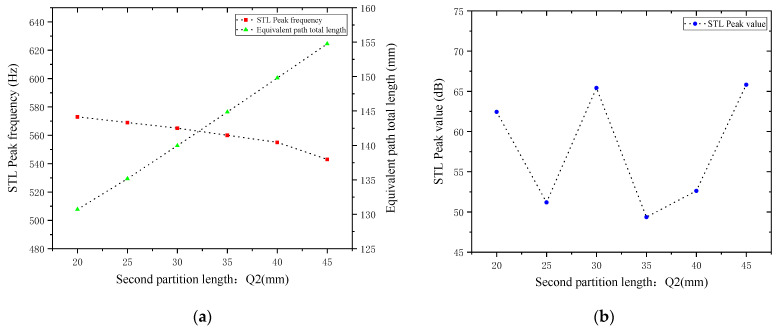
Comparison of noise reduction STL performance under different lengths of the second partition: (**a**) STL peak frequency; (**b**) STL peak magnitude.

Similarly, while keeping other parameters unchanged, the length of the second partition $Q_{2}$ was set to 20, 25, 30, 35, 40, and 45 mm. The corresponding sound transmission loss curves under these parameters are shown in Figure 16, and the comparative curves of noise reduction performance are shown in Figure 17.

As can be seen from Figure 16 and Figure 17, changes in $Q_{2}$ also affect both the STL peak frequency and magnitude. With an increase in $Q_{2}$, the total equivalent path length increases, causing the peak frequency to shift toward lower frequencies, and the shifting rate gradually accelerates, with a sharp drop in resonance frequency in the 40–45 mm range. Compared with the first partition, $Q_{2}$ results in a smaller decrease in the STL peak frequency, indicating that the first partition plays a primary regulatory role, while the second partition functions as a fine-tuning element. When $Q_{2}$ is 20, 30, and 45 mm, the STL peak magnitude exceeds 60 dB, and in other cases, it also exceeds 45 dB. Therefore, when setting the length of the second partition, it should be reasonably adjusted to exert its fine-tuning effect and optimize the noise reduction performance.

### 4.4. Influence of Internal Partition Positions

**Figure 18:**
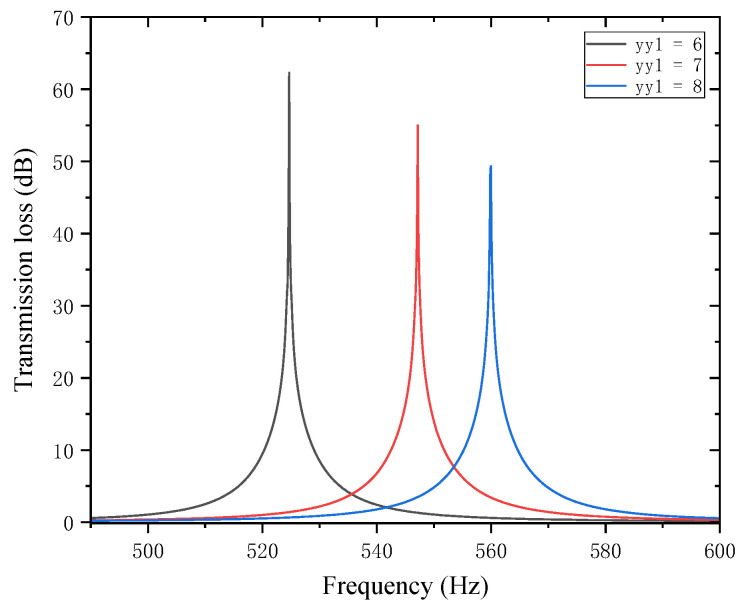
Influence of different positions of the first partition on the sound transmission loss of acoustic metamaterial mufflers.

**Figure 19:**
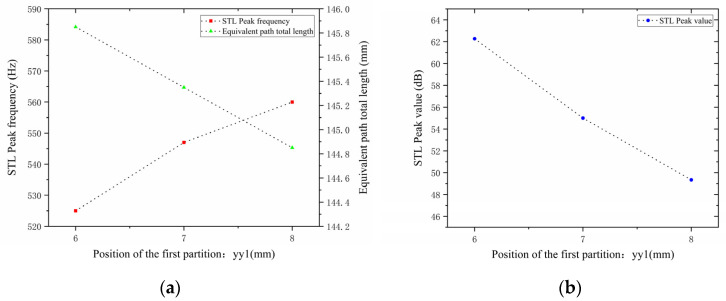
Comparison of noise reduction STL performance under different positions of the first partition: (**a**) STL peak frequency; (**b**) STL peak magnitude.

This section investigates the influence of the positions of two partitions in the perforated tortuous-characteristic acoustic metamaterial on the noise reduction performance of air ducts. When the positions of the internal partitions change, the volume of each chamber changes accordingly, which is equivalent to altering the internal structure of the muffler. This further affects the sound wave transmission path and resonance frequency, and thus the peak frequency of sound transmission loss may shift. In other words, the movement of partitions changes the equivalent length of the channel, thereby altering the resonance frequency. The movement of partitions also changes the cross-sectional area of each channel, which may enhance or weaken the formation of standing waves at certain frequencies, affect the energy dissipation of sound waves in the channel, alter the impedance matching of the muffler, and influence the reflection and transmission coefficients of sound waves. With other structural parameters of the perforated tortuous-characteristic acoustic metamaterial kept unchanged, the position of the first partition $yy_{1}$ was adjusted to 6, 7, and 8 mm, and the corresponding sound transmission loss curves of the acoustic metamaterial under these parameters were calculated. The amplitude–frequency characteristic curves are shown in Figure 18 below, and the comparative curves of noise reduction performance are shown in Figure 19.

As can be seen from Figure 18 and Figure 19, changes in $yy_{1}$ affect both the STL peak frequency and magnitude. With an increase in $\;yy_{1}$, the total equivalent path length decreases, causing the peak frequency to shift toward higher frequencies, and the shifting rate gradually slows down—rising sharply in the 6–7 mm range and gently in the 7–8 mm range. Meanwhile, as $yy_{1}$ increases, the peak magnitude decreases, with the decreasing rate slowing down gradually. Therefore, when setting the position of the first partition, it should be reasonably adjusted to optimize the noise reduction performance.

**Figure 20:**
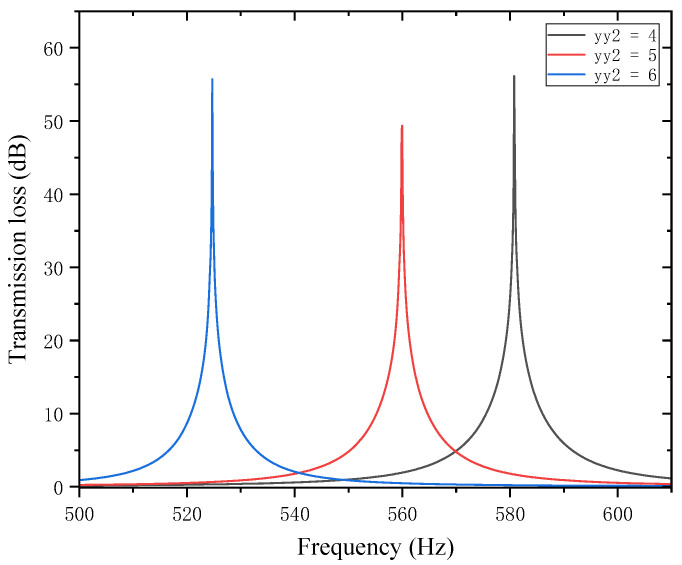
Influence of different positions of the second partition on the sound transmission loss of acoustic metamaterial mufflers.

**Figure 21:**
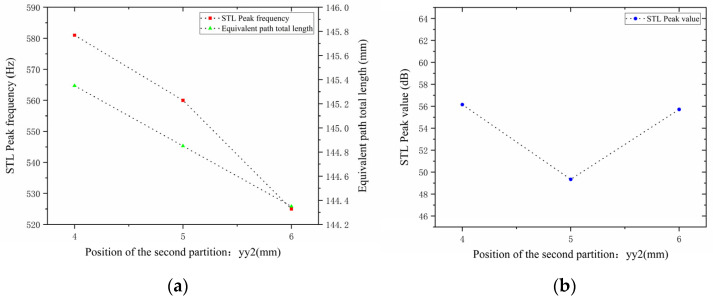
Comparison of noise reduction STL performance under different positions of the second partition: (**a**) STL peak frequency; (**b**) STL peak magnitude.

Similarly, with other structural parameters of the perforated tortuous-characteristic acoustic metamaterial kept unchanged, the position of the second partition $yy_{2}$ was adjusted to 4, 5, and 6 mm. The corresponding sound transmission loss curves of the acoustic metamaterial under these parameters were calculated, and the amplitude–frequency characteristic curves are shown in Figure 20 below. The comparative curves of noise reduction performance are shown in Figure 21.

As can be seen from Figure 20 and Figure 21, changes in the second partition position $yy_{2}$ affect both the position and magnitude of the STL peak frequency, with a pattern different from that of the first partition. As $yy_{2}$ increases, the total equivalent path length decreases, but the peak frequency shifts toward lower frequencies. This is because the movement of the partition alters the sound wave propagation path, causing changes in wavelength and perceived frequency. For each 1 mm increase in $yy_{2}$, the total equivalent path length decreases by 0.5 mm, and the rate at which its peak frequency shifts toward lower frequencies accelerates gradually. The STL peak magnitude first decreases and then increases, with all values exceeding 48 dB. Compared with the first partition, $yy_{2}\;$ has a greater impact on the variation range of the STL peak frequency and plays a primary regulatory role, while the first partition functions as a fine-tuning element. Therefore, when setting the position of the second partition, it should be reasonably adjusted to achieve the optimal noise reduction effect.

### 4.5. Influence of Trailing Plates Attached to Internal Partitions

#### 4.5.1. Length of the Trailing Plate

For the perforated tortuous-characteristic superstructure in this paper, each baffle end is connected with a trailing additional plate. Each trailing additional plate is divided into left and right trailing additional plates by taking the central axis of the baffle it is connected to as the boundary. Therefore, their influences on the noise reduction performance of the air duct are discussed separately. Changes in the length of the trailing additional plates will affect the total equivalent path length and cross-sectional area of the cavity channel, thereby regulating the noise elimination frequency.

**Figure 22:**
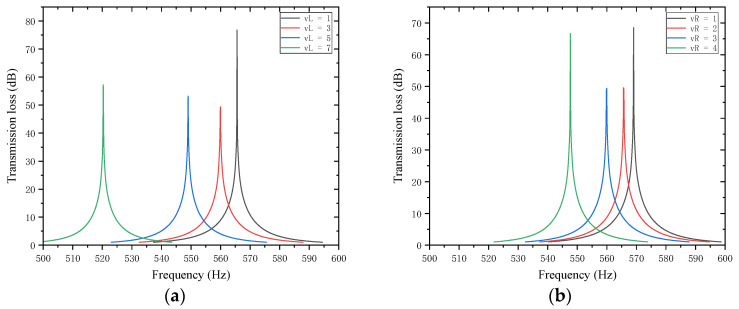
Influence of the length of trailing plates attached to the first partition on the sound transmission loss of acoustic metamaterial mufflers: (**a**) left trailing plate; (**b**) right trailing plate.

**Figure 23:**
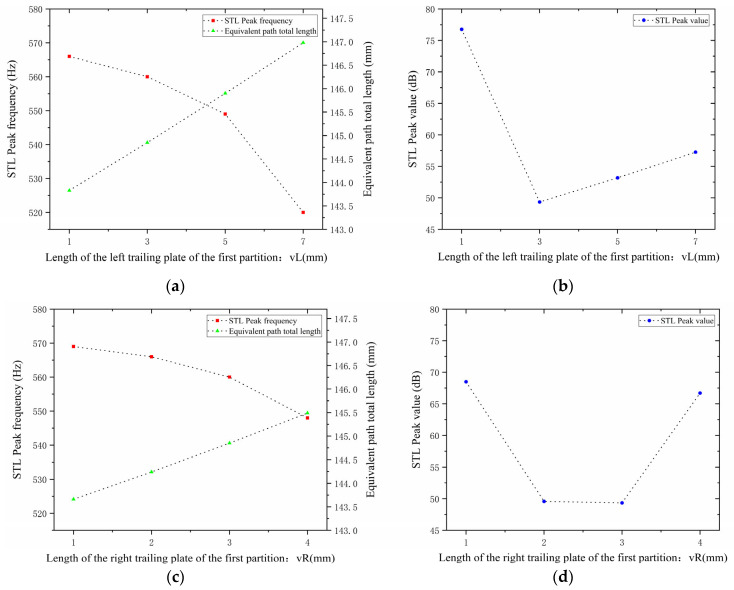
Comparison of noise reduction STL performance under different lengths of trailing plates attached to the first partition: (**a**) peak frequency of STL for the left trailing plate; (**b**) peak magnitude of STL for the left trailing plate; (**c**) peak frequency of STL for the right trailing plate; (**d**) peak magnitude of STL for the right trailing plate.

When studying the trailing plates attached to the first partition, with other parameters kept unchanged, the length of the left trailing plate $v_{L}$ was set to 1, 3, 5, and 7 mm, and the length of the right trailing plate $v_{R}$ was set to 1, 2, 3, and 4 mm. The sound transmission loss curves of the acoustic metamaterial under these corresponding parameters were calculated, and the amplitude–frequency characteristic curves are shown in Figure 22 below, with the comparative curves of noise reduction performance shown in Figure 23.

As shown in Figure 22 and Figure 23, variations in the lengths $v_{L}$ and $v_{R}$ of the left and right trailing plates of the first partition affect the STL peak frequency and magnitude. When $v_{L}$ increases, the total equivalent path length increases, causing the peak frequency to shift toward lower frequencies with a gradually increasing shifting rate, while the STL peak magnitude first drops sharply and then rises slowly. When $v_{R}$ increases, the total equivalent path length increases, leading the peak frequency to shift toward lower frequencies, and the peak magnitude first decreases and then increases.

**Figure 24:**
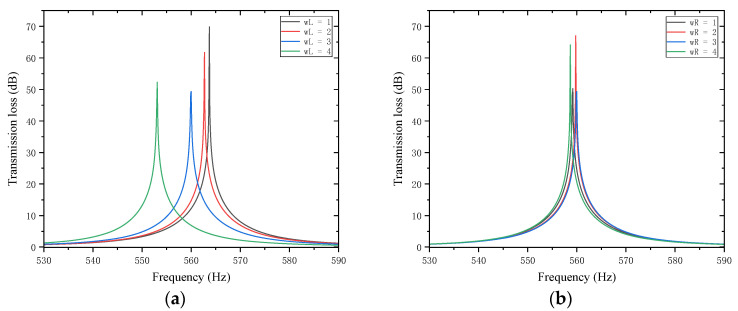
Influence of the length of trailing plates attached to the second partition on the sound transmission loss of acoustic metamaterial mufflers: (**a**) left trailing plate; (**b**) right trailing plate.

**Figure 25:**
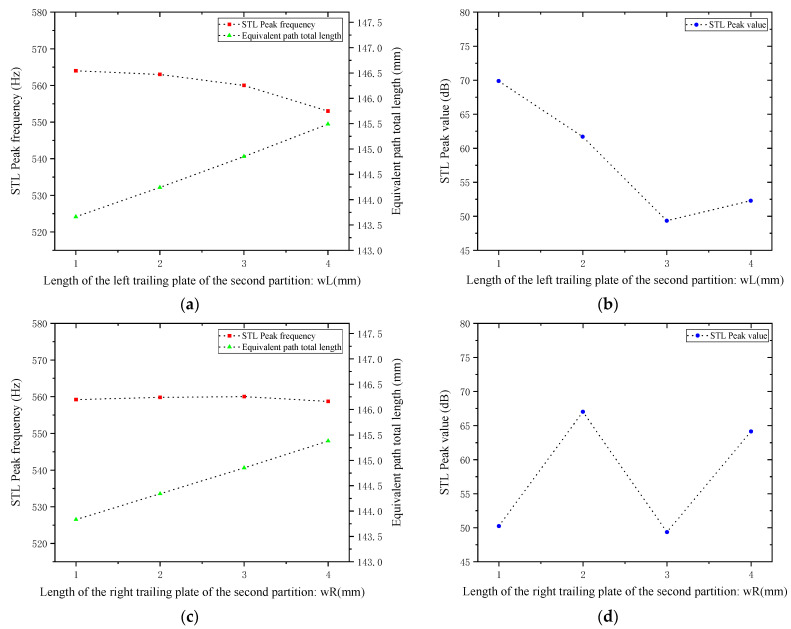
Comparison of noise reduction STL performance under different lengths of trailing plates attached to the second partition: (**a**) peak frequency of STL for the left trailing plate; (**b**) peak magnitude of STL for the left trailing plate; (**c**) peak frequency of STL for the right trailing plate; (**d**) peak magnitude of STL for the right trailing plate.

Similarly, for the trailing plates attached to the second partition, with other parameters kept unchanged, the length of the left trailing plate $w_{L}$ and the length of the right trailing plate $w_{R}$ were both adjusted to 1, 2, 3, and 4 mm. The sound transmission loss curves of the acoustic metamaterial under these corresponding parameters were calculated, and the amplitude–frequency characteristic curves are shown in Figure 24 below, with the comparative curves of noise reduction performance shown in Figure 25.

As can be seen from Figure 24 and Figure 25, increasing $w_{L}$ and $w_{R}$ results in an increase in the total equivalent path length, with the peak frequency shifting toward lower frequencies. Specifically, an increase in $w_{L}$ leads to a decrease in the peak frequency; in contrast, an increase in $w_{R}$ causes only negligible changes in the peak frequency. Notably, the STL peak magnitude reaches approximately 65 dB at 2 mm and 4 mm. Therefore, the length of the partition trailing appendage plates should be adjusted appropriately to optimize the sound attenuation performance.

#### 4.5.2. Thickness of the Trailing Plates

**Figure 26:**
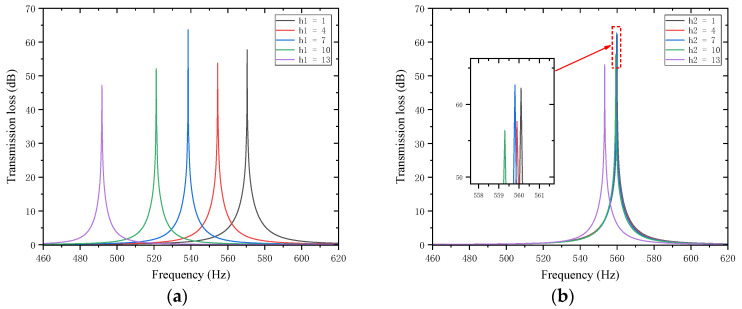
Influence of different thicknesses of partition trailing plates on the sound transmission loss of acoustic metamaterial mufflers: (**a**) trailing plates of the first partition; (**b**) trailing plates of the second partition.

**Figure 27:**
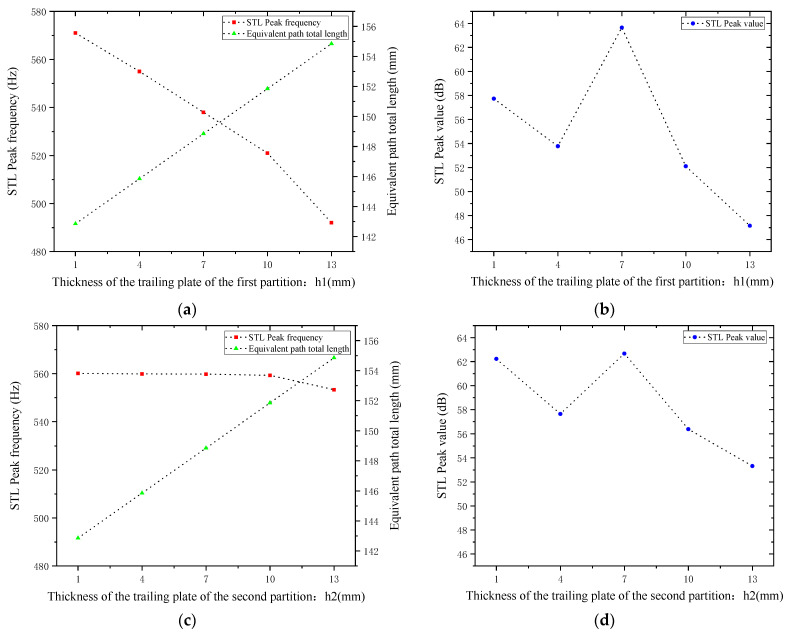
Comparison of noise reduction STL performance under different thicknesses of partition trailing plates: (**a**) Trailing plate of the first partition and STL peak frequency. (**b**) Trailing plate of the first partition and STL peak magnitude. (**c**) Trailing plate of the second partition and STL peak frequency. (**d**) Trailing plate of the second partition and STL peak magnitude.

Sound waves’ interference patterns and overall equivalent path length and resonance frequency are also impacted by variations in the thickness of the trailing plates that are attached to the first and second partitions. With other parameters kept unchanged, the thicknesses of the trailing plates attached to the first and second partitions $h_{1}\;$ and $h_{2}$ were adjusted to 1, 4, 7, 10, and 13 mm, respectively. The sound transmission loss curves of the acoustic metamaterial under these corresponding parameters were calculated, and the amplitude–frequency characteristic curves are shown in Figure 26 below, with the comparative curves of noise reduction performance shown in Figure 27.

As shown in Figure 26 and Figure 27, increasing $h_{1}$ and $h_{2}$ leads to an increase in the total equivalent path length, with the peak frequency shifting toward lower frequencies. The STL peak magnitude exhibits the same trend: it first decreases, then increases, and decreases again as the length increases. Specifically, the STL peak magnitude reaches the maximum when $h_{1}$ is 7 mm and when $h_{2}$ is 1 mm or 7 mm, which is attributed to intense interference and high acoustic impedance. Moreover, the peak magnitudes are almost all above 50 dB. Therefore, the thickness of the partition trailing appendage plates should be adjusted appropriately to achieve the optimal sound attenuation effect.

## 5. Conclusions and Prospects

This paper focuses on the low-frequency acoustic performance of a perforated tortuous-characteristic metamaterial air duct muffler. By combining theoretical modeling, finite element simulation, and structural parameter analysis, it reveals the mechanism behind its excellent noise reduction effect and verifies the feasibility and design advantages of this structure in engineering applications. The main conclusions of this paper are as follows:

1. Based on the idea of extending the sound wave propagation path, this paper designs a compact perforated tortuous-characteristic metamaterial unit cell with a thickness of only 50 mm. A complete theoretical model covering the perforated plate impedance, the equivalent path of the cavity, and the thermoacoustic loss mechanism is established, and the analytical expressions for the total surface acoustic impedance and transmission loss of the structure are derived.
2. This metamaterial structure is modeled using finite elements using the COMSOL simulation platform. There is a high degree of consistency between the theoretical calculations and the simulation results. Subsequent comparison with the conventional folded-channel metamaterial reveals that the two differ in effective bandwidth by 1 Hz, transmission loss by 1.157 dB, and resonance frequency by 38 Hz. This demonstrates good design effectiveness and confirms that the suggested structure has more notable benefits in terms of improving transmission loss and expanding bandwidth in the low-frequency range.
3. The influence laws of important structural parameters for acoustic performance, including perforation diameter, hole number, baffle size, and placement, are systematically indicated through parametric analysis. This lays the foundation for the wider use of broadband mufflers and theoretically supports the engineering optimization design of such metamaterial structures.

The limitations and prospects of this paper are as follows:

1. Existing studies are mainly based on ideal acoustic environments and have not fully considered multi-physics field coupling effects such as airflow velocity, temperature gradient, and turbulent disturbance in actual air ducts. In the future, a theoretical model involving airflow–acoustics–thermal field coupling can be established. Through numerical simulation and experimental testing, the influence laws of different environmental parameters for the muffler’s sound transmission loss (STL) can be analyzed.
2. The current research focuses on the unit cell structure, and the effective noise elimination frequency band still has limitations. In future studies, a multi-cell array structure can be designed based on the idea of “segmented regulation and frequency band complementarity”: by arranging unit cells with different hole diameters, baffle lengths, and trailing plate sizes according to specific rules, the low-frequency noise reduction performance can be expanded using the multi-resonance peak superposition effect.
3. The current research mainly focuses on theoretical modeling and numerical simulation, revealing the influence law of the new structure of this noise-reducing metamaterial on its acoustic performance at the mechanism level. Future research needs to carry out prototype processing and testing: 3D printing technology will be used to fabricate muffler samples, an air duct noise testing platform will be built in a semi-anechoic chamber, and the STL curves measured in experiments will be compared with theoretical and simulation results to further verify the practical effectiveness of the structural design.

## Figures and Tables

**Table 1:** The meaning of formula symbols.

$R_{s}$: Acoustic resistance	$Y_{s}$: Acoustic reactance	$Z_{ckb}$: Acoustic characteristic impedance of the perforated plate structure	$Z_{q}^{L1}$: Surface acoustic characteristic impedance at the first inlet
$\varphi_{0}$: Correction coefficient	$S_{0}$: Cross-sectional area of the perforated panel	$S_{i}$: Cross-sectional area of different chamber channels	$\rho_{0}$: Density under standard ambient temperature conditions
μ: Dynamic viscosity coefficient	ω: Angular frequency	$t_{c}$: Axial length of the micro-short tube	$r_{m}$: Acoustic resistance
$m_{m}$: Acoustic mass	$k_{r}$: Acoustic resistance constant	$k_{m}$: Acoustic mass constant	$c_{0}$: Speed of sound
σ: Perforation rate	$k_{0}$: Wavenumber in air	$Z_{0}$: Acoustic characteristic impedance in air	$\xi_{l}$: Radiation correction length
$d_{crv1}$: Inlet width	$k_{qti}$: Effective wavenumber of the cavity	$l_{qti}$: Approximate effective length of each cavity channel in the metastructure	$Z_{qti}$: Channel characteristic impedance
$\rho_{cpi}$: Effective complex density	$K_{cpi}$: Complex bulk modulus of gas	γ: Specific heat ratio	$\kappa_{0}$: Thermal conductivity of air
$C_{\psi_{0}}$: Specific heat capacity at constant volume	N: Number of effective channels in the cavity	D: Cross-section of the internal cavity of the air duct	H: Cross-section of the internal cavity of the air duct
M: Thickness of the metastructure			

**Table 2:** Simulation parameters of air.

Material Parameters	Parameter Values	Parameter Units
Speed of sound	343.204	m/s
Density	1.204	kg/m^3^
Thermal conductivity	2.978 × 10^−5^	W/(m·K)
Dynamic viscosity	1.814 × 10^−5^	Pa·s
Bulk viscosity	1.088 × 10^−5^	Pa·s
Specific heat capacity at constant pressure	1005	J/(kg·K)
Specific heat ratio	1.4	1

**Table 3:** Dimensional values of the dual-baffle perforated meandering metamaterial structure.

**Diameter of perforations in the perforated plate**	**Number of perforations in the perforated plate**	**Length of the first baffle**	**Length of the left trailing plate attached to the first baffle**	**Length of the right trailing plate attached to the first baffle**	**Thickness of the trailing plates attached to the first baffle**
$d_{c}=$ 3 mm	m= 3	$Q_{1}=$ 35 mm	$v_{L}=$ 3 mm	$v_{R}=$ 3 mm	$h_{1}=$ 3 mm
**Position of the first baffle**	**Length of the second baffle**	**Length of the left trailing plate attached to the second baffle**	**Length of the right trailing plate attached to the second baffle**	**Thickness of the trailing plates attached to the second baffle**	**Position of the second baffle**
$yy_{1}=$ 8 mm	$Q_{2}=$ 35 mm	$w_{L}=$ 3 mm	$w_{R}=$ 3 mm	$h_{2}=$ 3 mm	$yy_{2}=$ 5 mm

**Table 4:** Analytical expressions for effective cross-sectional areas of cavity channels.

Cross-Sectional Area	Analytical Expression
$S_{1}$	$yy_{1}\times D$
$S_{2}$	$\left(yy_{1}+0.5t_{q}-v_{L}\right)\times D$
$S_{3}$	$\left(M-t_{q}-t_{c}-Q_{1}-h_{1}\right)\times D$
$S_{4}$	$\left(D-yy_{1}-yy_{2}-1.5t_{q}-v_{R}\right)\times D$
$S_{5}$	$\left(D-yy_{1}-yy_{2}-2t_{q}\right)\times D$
$S_{6}$	$\left(D-yy_{1}-yy_{2}-1.5t_{q}-w_{L}\right)\times D$
$S_{7}$	$\left(M-t_{q}-t_{c}-Q_{2}-h_{2}\right)\times D$
$S_{8}$	$\left(yy_{2}+0.5t_{q}-w_{R}\right)\times D$
$S_{9}$	$yy_{2}\times D$

**Table 5:** Numerical values of effective cross-sectional areas of cavity channels.

**Cross-Sectional Area**	$S_{1}$	$S_{2}$	$S_{3}$	$S_{4}$	$S_{5}$	$S_{6}$	$S_{7}$	$S_{8}$	$S_{9}$
**Value (mm^2^)**	160	110	240	50	100	50	240	50	100

**Table 6:** Analytical expressions for equivalent path lengths of cavity channels.

Channel Equivalent Path	Analytical Expression
$l_{qt1}$	$\sqrt{{Q_{1}}^{2}+{\left(\frac{1}{2}v_{L}-\frac{1}{4}t_{q}\right)}^{2}}$
$l_{qt2}$	$\frac{1}{2}\left(M-Q_{1}-t_{q}-t_{c}+h_{1}\right)$
$l_{qt3}$	$\frac{1}{2}\left(D-yy_{2}+v_{L}+v_{R}-t_{q}\right)$
$l_{qt4}$	$\frac{1}{2}\left(M-Q_{1}-t_{q}-t_{c}+h_{1}\right)$
$l_{qt5}$	$\sqrt{{\left(Q_{1}+Q_{2}-M+t_{q}+t_{c}\right)}^{2}+{\left[\frac{1}{2}\left(v_{R}+w_{L}-t_{q}\right)\right]}^{2}}$
$l_{qt6}$	$\frac{1}{2}\left(M-Q_{2}-t_{q}-t_{c}+h_{2}\right)$
$l_{qt7}$	$\frac{1}{2}\left(D-yy_{1}+w_{L}+w_{R}-t_{q}\right)$
$l_{qt8}$	$\frac{1}{2}\left(M-Q_{2}-t_{q}-t_{c}+h_{2}\right)$
$l_{qt9}$	$\sqrt{{Q_{2}}^{2}+{\left(\frac{1}{2}w_{R}-\frac{1}{4}t_{q}\right)}^{2}}$

**Table 7:** Numerical values of equivalent path lengths and total equivalent path length for cavity channels.

**Effective Path**	$l_{qt1}$	$l_{qt2}$	$l_{qt3}$	$l_{qt4}$	$l_{qt5}$	$l_{qt6}$	$l_{qt7}$	$l_{qt8}$	$l_{qt9}$	$l_{qt-all}$
**Value (mm)**	35.02	9.00	10.00	9.00	20.31	9.00	8.50	9.00	35.02	144.85

**Table 8:** Values of equivalent path length of each cavity channel and total equivalent path length.

**Effective Path**	$l_{ct1}$	$l_{ct2}$	$l_{ct3}$	$l_{ct-all}$
**Value (mm)**	42.74	35.51	42.61	120.85

## Data Availability

The original contributions presented in this study are included in the article; further inquiries can be directed to the corresponding authors.
